# Plexin-B2 and Plexin-D1 in Dendritic Cells: Expression and IL-12/IL-23p40 Production

**DOI:** 10.1371/journal.pone.0043333

**Published:** 2012-08-15

**Authors:** Eda K. Holl, Kelly E. Roney, Irving C. Allen, Erin Steinbach, Janelle C. Arthur, Adam Buntzman, Scott Plevy, Jeffrey Frelinger, Jenny P.-Y. Ting

**Affiliations:** 1 Department of Microbiology and Immunology, University of North Carolina at Chapel Hill, Chapel Hill, North Carolina, United States of America; 2 Lineberger Comprehensive Cancer Center, University of North Carolina at Chapel Hill, Chapel Hill, North Carolina, United States of America; 3 Department of Medicine, University of North Carolina Chapel Hill, Chapel Hill, North Carolina, United States of America; 4 Department of Immunobiology, University of Arizona, Tucson, Arizona, United States of America; Istituto Superiore di Sanità, Italy

## Abstract

Plexins are a family of genes (A,B,C, and D) that are expressed in many organ systems. Plexins expressed in the immune system have been implicated in cell movement and cell-cell interaction during the course of an immune response. In this study, the expression pattern of Plexin-B2 and Plexin-D1 in dendritic cells (DCs), which are central in immune activation, was investigated. Plexin-B2 and Plexin-D1 are reciprocally expressed in myeloid and plasmacytoid DC populations. Plasmacytoid DCs have high Plexin-B2 but low Plexin-D1, while the opposite is true of myeloid DCs. Expression of Plexin-B2 and Plexin-D1 is modulated upon activation of DCs by TLR ligands, TNFα, and anti-CD40, again in a reciprocal fashion. Semaphorin3E, a ligand for Plexin-D1 and Plexin-B2, is expressed by T cells, and interestingly, is dramatically higher on Th2 cells and on DCs. The expression of Plexins and their ligands on DCs and T cells suggest functional relevance. To explore this, we utilized chimeric mice lacking *Plxnb2* or *Plxnd1.* Absence of Plexin-B2 and Plexin-D1 on DCs did not affect the ability of these cells to upregulate costimulatory molecules or the ability of these cells to activate antigen specific T cells. Additionally, Plexin-B2 and Plexin-D1 were dispensable for chemokine-directed *in-vitro* migration of DCs towards key DC chemokines, CXCL12 and CCL19. However, the absence of either Plexin-B2 or Plexin-D1 on DCs leads to constitutive expression of IL-12/IL-23p40. This is the first report to show an association between Plexin-B2 and Plexin-D1 with the negative regulation of IL-12/IL-23p40 in DCs. This work also shows the presence of Plexin-B2 and Plexin-D1 on mouse DC subpopulations, and indicates that these two proteins play a role in IL-12/IL-23p40 production that is likely to impact the immune response.

## Introduction

Semaphorins and plexins were initially identified as key molecules in axon guidance during neuronal development [1], [2]. Semaphorins are classified into three different groups based on their origin and structural homology; invertebrate, vertebrate and viral semaphorins [3]. Plexin receptors are divided into two large groups, invertebrate and vertebrate, and further subdivided into four different families, A–D [4]. Although plexins are considered receptors for semaphorins, this view has been revised as semaphorins have been demonstrated to mediate signal transduction [5]–[8]. The interactions between semaphorins and plexins are varied. Semaphorins can interact with multiple plexins on a single cell type or across multiple cell types and vice versa [4]. Plexins and semaphorins control cell movement and migration and have been implicated in neural cell function, vasculature formation, and organ development [9]–[12].

Recent work has implicated plexins and semaphorins in the regulation of immune system [13]–[16]. Several plexins and semaphorins are expressed by both naïve and activated immune cells. Plexin-D1 and Semaphorin-3E are expressed in the thymus [12]; Plexin-A1 and Semaphorin-6D are expressed on DCs and T cells respectively [14], [17], [18]; Semaphorin-4A is expressed by Th1 polarized T cells and DCs [19]; Semaphorin-4D is expressed by T cells, DCs, and activated B cells [13], [20]–[24]; Plexin-A4 is expressed by T cells, B cells and DCs [16]; and Plexin-C1 is also expressed by DCs [15]. The wide distribution of plexins and semaphorins across immune system cells and environments suggest that they function in immune system development and response.

The function of plexins and semaphorins on DCs has not yet been fully characterized. Plexin-A1 expression on DCs is required for proper T-cell activation and DC migration [14], [18]. Semaphorin-6D, a known ligand for Plexin-A1, is expressed on activated T cells and is required for late-phase T cell proliferation [17]. Mice deficient in Plexin-A4 develop exacerbated MOG-induced experimental autoimmune encephalomyelitis (EAE) and defective inflammatory cytokine production [16], [25]. Semaphorin-4D maintains B-cell homeostasis and facilitates humoral immune responses [22]. The functions of plexins and semaphorins in cell to cell communication demonstrate their importance in the immune response.

To date, research regarding Plexin-D1 and Plexin-B2 in the immune system has been limited. In other systems Plexin-D1 partners with two different semaphorin molecules: Semaphorin-3E and Semaphorin-4A [11], [26]. Plexin-B2 has been found to have several semaphorin ligands including Semaphorin-3E, Semaphorin-4A, Semaphorin-4C, and Semaphorin-4D [27]–[30]. Plexin-D1 was recently shown to be expressed by double positive thymocytes and facilitate their migration from the cortex into the medulla [12]. Plexin-B2 is expressed on T-dependent germinal center B cells but not T-independent germinal center B cells, though the physiological consequence of this increase in expression are unknown [31]. Studies of development in the model organism zebrafish have shown that Plexin-B2 and Plexin-D1 both impact sprouting, but in different ways. Plexin-B2 deficiency results in delayed migration of sprouting angioblast while Plexin-D1 deficiency results in early angioblast sprouting [27]. These findings show that zebra fish Plexin-B2 and Plexin-D1 both function in the proper timing of cell homing, but their functional effects are exerted indifferent ways.

This study reports that Plexin-B2 and Plexin-D1 are differentially expressed in DCs and shows that this expression can be modulated by a number of immune activators. To address the role of Plexin-B2 and Plexin-D1 in DC development and function, *Plxnd1^−/−^ and Plxnb2^−/−^* mice were utilized. Due to the embryonic lethality of these mice, chimeric mice created by fetal liver transplantation were utilized as previously described [32]. I*n vitro* and *in vivo* approaches to examine the direct effect of Plexin-B2 and Plexin-D1 on DCs were employed. These studies show that DCs lacking Plexin-B2 and Plexin-D1 were capable of inducing a normal CD4+ T cell response, migrating properly towards chemokines and accumulating in the spleen similar to wildtype cells. Additionally, *Plxnb2^−/−^* and *Plxnd1^−/−^* DCs secrete normal amounts of TNFα and IL-6. However, both *Plxnb2^−/−^* and *Plxnd1^−/−^* DCs are hyper-responsive in their secretion of IL-12/IL-23 p40 at the steady state. This suggests that Plexin-B2 and Plexin-D1 are negative regulators of IL-12/IL-23p40 response, demonstrating both plexins may function in the same pathway in DC. Modulation of this important pathway may be controlled by the targeting both Plexin-B2 and Plexin-D1.

## Results

### Plexin-B2, Plexin-D1, and Semaphorin-3E Expression in Immune Cells

**Figure 1:**
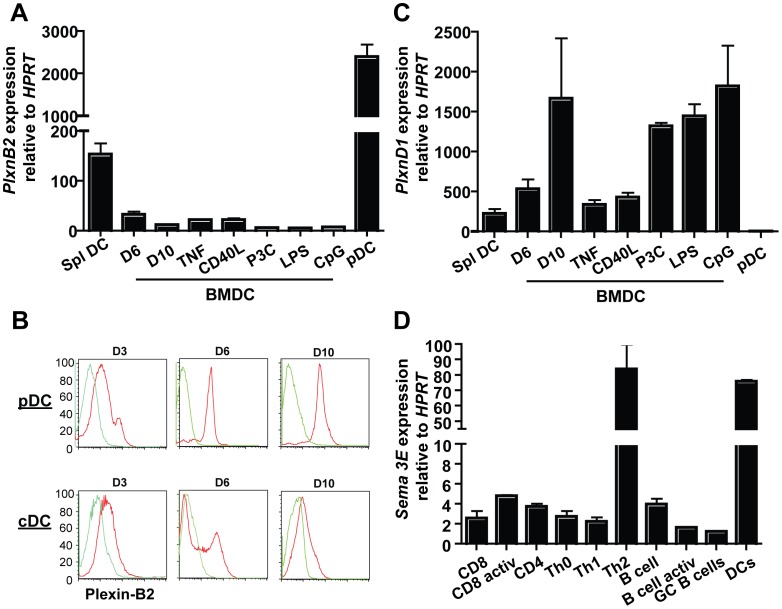
Plexin-B2, Plexin-D1, and Semaphorin-3E expression. (A) Expression of *Plxnb2* in splenic DCs, BMDCs at day 6 (D6) and day 10 (D10), D10 post 16 hour activation by TNF (20 ng/ml), CD40L (1 µg/ml), TLR ligands P3C (1 µg/ml), (LPS (1 µg/ml), CpG (4 µg/ml), and plasmacytoid DCs (pDCs) as measured by real-time PCR. Data are representative of three independent experiments. (B) Expression of *PlxnB2* in BM-derived pDCs and cDCs at D3, D6, and D10. Green lines indicate IgG control antibody staining, red histograms are *Plxnb2* antibody staining. (C) Expression of *Plxnd1* in sDCs, BM-derived DCs D6, D10, post activation, and pDC as measured by real-time PCR. (D) Expression of *Semaphorin3E* in sorted naïve and activated T cell and B cell populations, and DCs. Data are representative of 3 independent experiments.

Expression patterns of individual plexins in the immune system during cell maturation or activation have been reported in the literature. Prior work primarily focused on the expression of Plexin-B2 and Plexin-D1 on lymphocytes. Plexin-B2 is expressed on B cells from T cell dependent germinal centers but not T independent germinal centers [31]. Plexin-D1 is expressed by thymocytes and further down-regulated with T cell maturation as well as by activated B cells [12], [32]. However the expression of these two genes in cells of the myeloid-monocytic lineage has not been explored. Studies in this paper extended Plexin-B2 and Plexin-D1 expression studies to include another immune cell type, DCs. DCs are required for T cell priming in the secondary lymphoid organs. Plexin-B2 and Plexin-D1 is expressed on cells sorted by flow cytometry which represent splenic myeloid DCs (mDCs) (CD11b^+^CD11c^+^) (Figure1A, B, and C). Plexin-B2 is expressed by bone marrow derived DCs (BMDCs) that were treated with GM-CSF and IL-4 for 6 days (D6), while cells treated with the same cytokines for 10 days (D10) show decreased expression, and then increases slightly with the addition of TNFα and CD40L treatment (Figure 1A). Plexin-B2 expression on mDCs is not increased by treatment with toll-like receptor (TLR) ligands (P3C, TLR1/2), lipopolysaccharide (LPS, TLR4), or CpG (TLR9). Plexin-B2 is also highly expressed by plasmacytoid DCs (CD11c^+^B220^+^mPDCA1^+^) (Figure 1A, B).

The expression pattern of Plexin-B2 is in contrast with the expression pattern of Plexin-D1. It increases during the maturation of BMDCs and with treatment by TLR ligands P(3)C, LPS, and CpG (Figure 1C). Plexin-D1 expression is not increased in response to treatment with TNFα or CD40L, and is expressed at a negligible level on plasmacytoid DCs. Both Plexin-B2 and Plexin-D1 are expressed on splenic *ex vivo* DCs (Figure 1A, C).

Expression of Plexin-B2 throughout DC maturation was confirmed at the protein level using a monoclonal antibody against Plexin-B2 (Figure 1B). In conventional DCs that are produced with GM-CSF and IL-4, the data shows that expression of Plexin-B2 is bimodal at day 6, but singular with lower peak expression at day 10. In plasmacytoid DCs that are matured with Flt3L at day 6 and 10, Plexin-B2 is highly expressed. These data support the differential cDNA expression pattern of Plexin-B2. Antibodies to detect Plexin-D1 effectively are not commercially available; therefore, we did not determine expression of Plexin-D1 at the protein level.

Studies have shown that the predominant Plexin-D1 partner in the immune system is Semaphorin-3E, although the specific cell type providing the ligand is unknown [11], [12]. These studies show that Semaphorin-3E is expressed in the thymic medulla where it creates a gradient that is responsible for migration of Plexin-D1 expressing thymocytes from the cortex into the medulla [12]. The immune system binding partner for Plexin-B2 is unknown. However, in zebra fish angioblast, Semaphorin-3E is the ligand for Plexin-B2, and Plexin-D1 antagonizes the Plexin-B2/Semaphorin-3E pathway. Upon observing the opposing expression patterns of Plexin-B2 and Plexin-D1 in cDCs, we hypothesized that Semaphorin-3E may also be present on DCs. Semaphorin-3E expression was analyzed in a number of immune cells (Figure 1D). The data show that Semaphorin-3E is minimally detected in naïve and activated T and B cell populations. However, Semaphorin-3E is highly expressed on Th2 skewed T cells and splenic cDCs. The expression pattern of Semaphorin-3E suggests that partnering of this protein with Plexin-B2 and Plexin-D1 during the course of an immune response may be important for T cell function.

### Plxnb2^−/−^ and Plxnd1^−/−^ do not Affect DC Maturation

**Figure 2:**
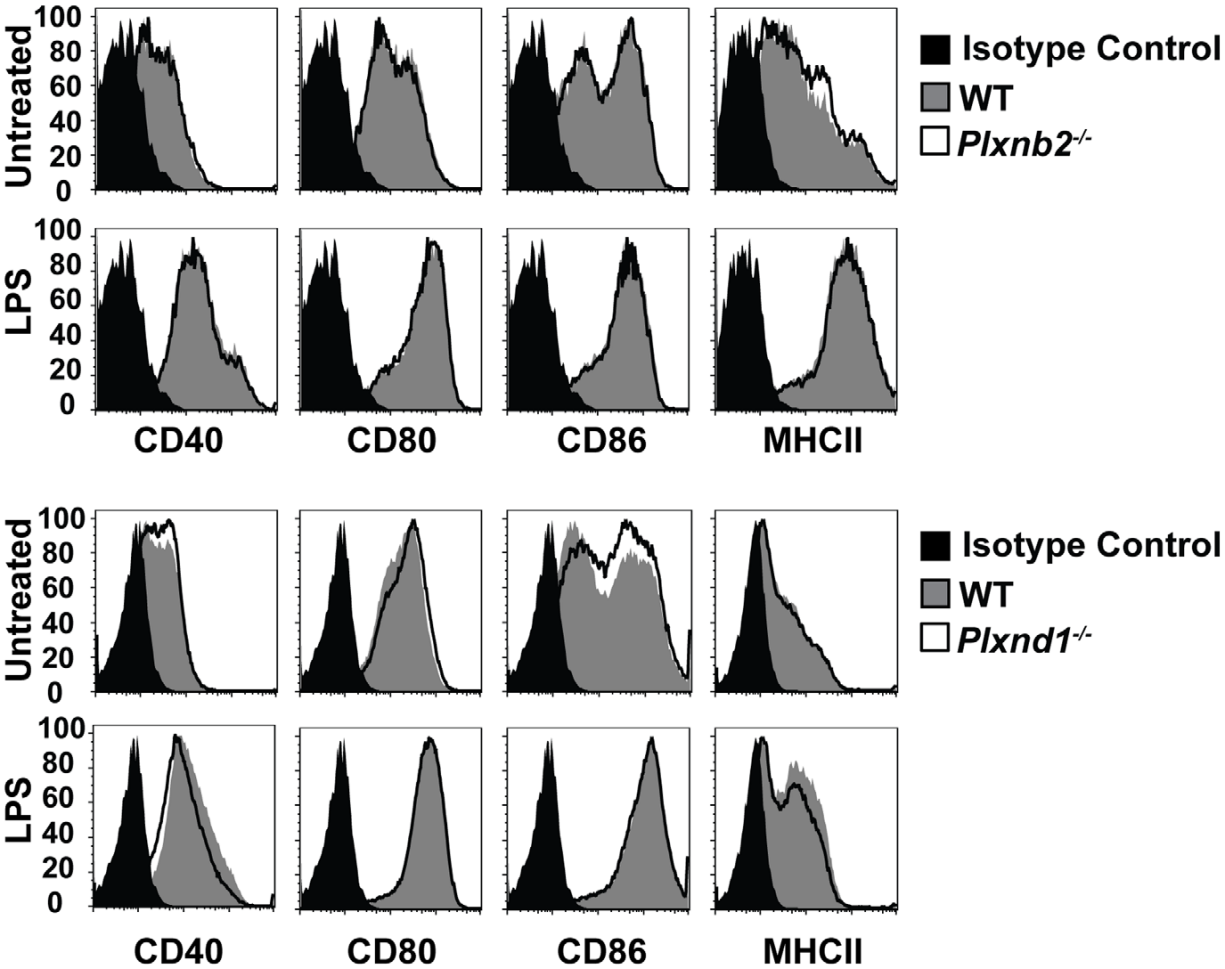
DCs maturation is not affected in the absence of *Plxnb2* and *Plxnd1*. *Plxnb2^−/−^* and *Plxnd1^−/−^* DCs are able to upregulate cell surface receptors. DCs were derived in the presence of GM-CSF and IL4 from the bone marrow of mice reconstituted with *Plxnb2−/−, Plxnd1^−/−^,* and wild type fetal liver cells. DCs were then cultured in the presence of LPS and cell surface receptor expression was assessed 24 hours later using flow cytometry. Data are representative of 3 independent experiments. n = 6 mice per group.

To further assess function of Plexin-B2 and Plexin-D1, *Plxnb2^−/ −^* and *Plxnd1^−/−^* DCs were studied. *Plxnb2^−/−^* and *Plxnd1^−/−^* mice die shortly after birth, and therefore fetal liver chimeric mice were utilized to study Plexin-B2 and Plexin-D1 in the immune system. Lethally irradiated wild type congenic mice were reconstituted with hematopoietic cells from E14 fetal livers of Plxnb2*^−/−^, Plxnd1^−/−^*, or wild type siblings. *Plxnb2^−/−^* and *Plxnd1^−/−^* DCs were derived *in vitro* in the presence of GM-CSF and IL-4. BMDCs from *Plxnb2^−/−^, Plxnd1^−/−^*, and wild type chimeric mice were then stimulated with LPS to determined expression of co-stimulatory molecules on the DCs. Surface levels of CD40, CD80, CD86, and I-Ab were equivalent between wild type, *Plxnb2^−/−^* and *Plxnd1^−/−^* mice at both the basal level and post activation indicating that these two plexins do not affect DC maturation (Figure 2).

### Plxnb2^−/−^ and Plxnd1^−/−^ DCs Stimulate T Cells Similarly to Wild Type

DCs are proficient antigen presenting cells required for proper T cell selection in the thymus and activation of naïve T cells in the periphery [33], [34]. DCs take up antigen and present it to T cells in the context of MHC molecules [34]. Upon cognate antigen encounter, T cells proliferate, release a series of cytokines, and function as cytotoxic T-lymphocytes or helper T cells (Th) [34].

**Figure 3:**
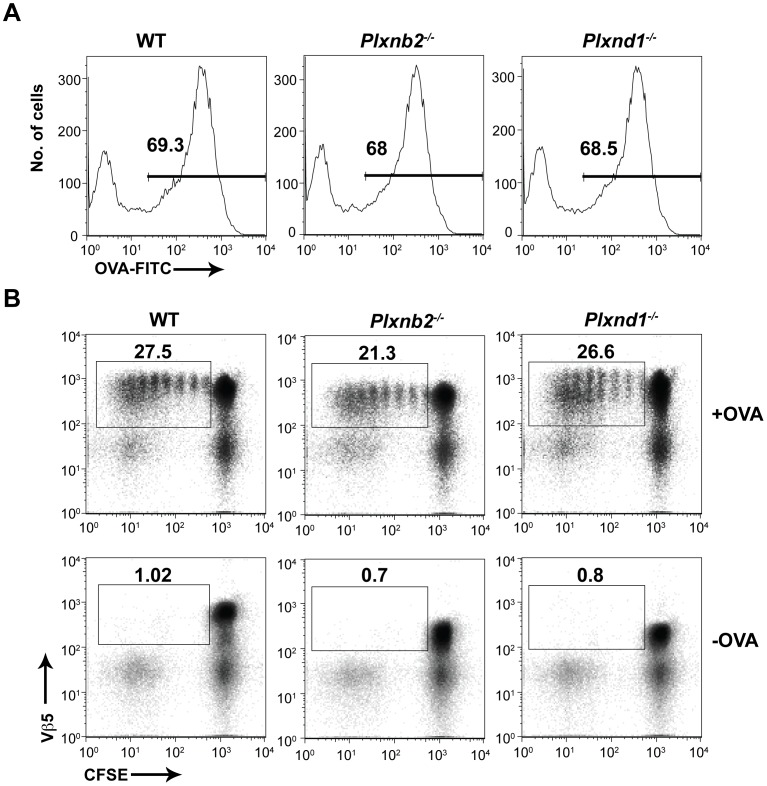
*Plxnb2^−/−^* and *Plxnd1^−/−^* DCs efficiently stimulate antigen specific T cells. (A) OVA uptake for *Plxnb2^−/−^, Plxnd1^−/−^,* and wild type DCs. DCs were isolated from spleens of mice reconstituted with *Plxnb2^−/−^, Plxnd1^−/−^,* and wild type fetal liver cells and cultured in the presence of OVA-FITC for 30 minutes. OVA uptake was assessed by flow cytometry. Data are representative of 2 independent experiments. n = 6 mice per group. (B) DCs were isolated from spleens of mice reconstituted with *Plxnb2^−/−^, Plxnd1^−/−^,* and wild type fetal liver cells. DCs were then co-cultured with OTII-specific T cells in the presence of OVA and T cell proliferation was assessed by CFSE dilution 72 hours later using flow cytometry. Data are representative of 3 independent experiments. n = 9 mice per group.

The ability of *Plxnb2^−/−^* and *Plxnd1^−/−^* DCs to take up ovalbumin (OVA) protein was assessed *in vitro*. DCs were cultured for 2 hours in the presence of FITC-labeled OVA protein. The amount of OVA taken up by DCs was assessed by flow cytometry. *Plxnb2^−/−^*, *Plxnd1^−/−^* and wild type DCs were able take up antigen equivalently as shown by the level of mean fluorescence intensity of the analyzed DCs (Figure 3A).

To determine the role of Plexin-B2 and Plexin-D1 in DCs, we performed an *in vitro* antigen presentation assay using transgenic T cells specific for OVA peptide 323–339, OTII T cells. Freshly isolated carboxyfluorescein succinimidyl ester (CFSE)-labeled OTII T cells were cultured in the presence of OVA protein-pulsed *Plxnb2^−/−^*, *Plxnd1^−/−^*, and wild type splenic DCs for three days. Non OVA-pulsed DCs were used as a negative control. Proliferation of Vβ5^+^ OTII T cells was assessed by flow cytometry. As shown in Figure 3B, *Plxnb2^−/−^* and *Plxnd1^−/−^* DCs are capable of stimulating T cells similarly to wild type. These data suggests that neither Plexin-B2 nor Plexin-D1 play critical roles during the *in vitro* activation of CD4^+^ T cells by DCs.

### Plexin-B2 and Plexin-D1 do not Affect the Migration of DCs

**Figure 4:**
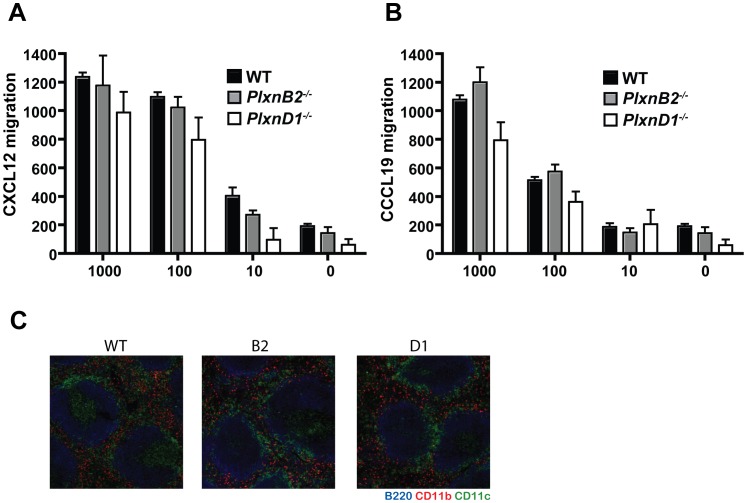
*Plxnb2^−/−^* and *Plxnd1^−/−^* DCs migrate similarly to wild type control towards chemokine gradients. (A) Purified wildtype (black bar), *Plxnb2^−/−^* (grey bar), and *Plxnd1^−/−^* (open bar) DCs were placed in upper wells and subjected to *in-vitro* migration assays in the presence of medium alone, CXCL12 and CCL19. Migrated cells were quantified by toxilight (Lonza, Basel, Switzerland) according to the manufacturers instructions and normalized to a standard curve n = 6–7 mice per group. (B) Five µm sections of spleens from wildtype, *Plxnb2^−/−^* and *Plxnd1^−/−^* mice were labeled with B220-AF350 (blue), CD11b-PE (red) and CD11c-FITC (green). FITC signal was amplified using anti-FITC-AF488. Images were acquired using a Zeiss Axiovert 200 M confocal immunofluorescent microscope. n = 3 mice per group.

Plexins and semaphorins have been implicated in migration of many different cell types including neuronal, endothelial, and immune cells [7]. In zebra fish angioblast cells, knockdown of Plexin-B2 or its ligand Semaphorin-3E yields delayed sprouting of intersegmental (ISV) angioblast during development [27]. Knockdown of Plexin-D1 in zebra fish embryos results in an opposite effect of early ISV sprouting [27]. In the mouse nervous system, *Plxnb2^−/−^* animals show defects in neuronal cell homing that result in neural tube closure defects and cerebellum disorganization [35]. Plexin-D1 is required for endothelial cell patterning [11] as well as migration of DP thymocytes from the cortex into the medulla during thymic maturation in mouse [12]. Therefore we investigated the ability of *Plxnb2^−/−^* and *Plxnd1^−/−^* DCs to migrate towards chemokines that are associated DC homing to the lymph nodes, CXCL12 and CCL19 [18]. DCs were placed in the top chamber of transwell migration chambers. Cytokines CXCL12 (Figure 4A), CCL19 (Figure 4B), or media alone as a control were place in the bottom of the transwell chambers. We observed that at the 10 ng, 100 ng, and 1000 ng concentrations of cytokines and with the media control *Plxnb2^−/−^* DCs migrated similarly to wild type. *Plxnd1^−/−^* DCs migrated less efficiently towards CXCL12 compared to wild type (Figure 4A), although the trend did not reach statistical significance. This trend was not observed during migration towards CCL19 (Figure 4B). To further investigate the migration and homing capabilities of DCs deficient in Plexin-B2 or Plexin-D1, DCs in the spleens of *Plxnb2^−/−^*, *Plxnd1^−/−^*, and wild type reconstituted mice were visualized using immunoflourescent staining. In Figure 4C, the data show that CD11b^+^ macrophages, CD11c^+^ DCs, and B220^+^ B cells were present in similar architecture in *Plxnb2^−/−^*, *Plxnd1^−/−^*, and wild type spleens. This data shows that Plexin-B2 and Plexin-D1 did not affect migration towards lymph node homing cytokines or the architecture of DCs, macrophages, or B cells to the spleen.

### Plexin-B2 and Plexin-D1 Affect the Basal IL-12/IL-23p40 Expression

**Figure 5:**
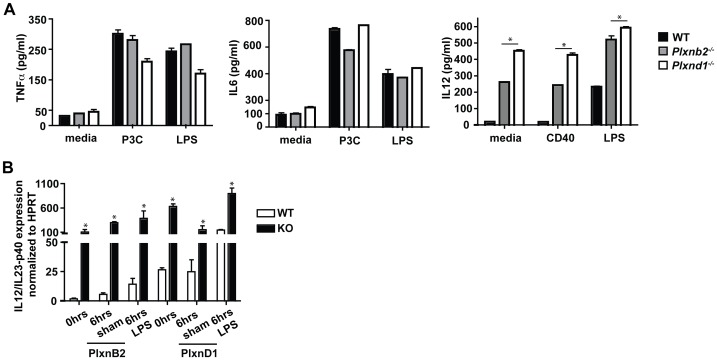
*Plxnb2^−/−^* and *Plxnd1^−/−^* DC time-course of IL-12/IL-23p40 cDNA. (A) *Plxnb2^−/−^* and *Plxnd1^−/−^* DCs are able to produce inflammatory cytokines in response to TLR stimuli and anti-CD40. DCs were cultured in the presence of LPS, P3C, or anti-CD40 for 24 hours. Culture supernatants were assessed for production of TNFα, IL-6, and IL-12/IL-23 p40 by ELISA. Data are representative of 3 independent experiments. n = 3–4 mice per group. *p<0.01. (B) DCs were isolated from the spleens of *Plxnb2^−/−^* and *Plxnd1^−/−^* mice and cultured in the presence of LPS for 6 hours. Cells were collected and mRNA was isolated to determine mRNA levels of IL-12/IL-23 p40 by real-time PCR. Data are representative of 3 independent experiments. n = 3–4 mice per group. *p<0.01.

To characterize DC activation cytokine profile experiments of the common DC cytokines IL-6, TNFα, and IL-12/IL-23p40 were performed. Supernatants were collected from treatment of DC cultures with LPS, P3C, and anti-CD40 antibody and followed by ELISAs to determine amount of cytokine released from the DCs. As shown in Figure 5A, we determined that levels of IL-6 and TNFα cytokines released in culture supernatants 24 hours post stimulation were equivalent between *Plxnb2^−/−^*, *Plxnd1^−/−^*, and wild type DCs. However, we observed that in unstimulated, CD40L stimulated, and LPS stimulated DCs, protein levels of IL-12/IL-23p40 are higher in both *Plxnb2^−/−^* and *Plxnd1^−/−^* DCs when compared to wild type cells. Additionally, a time course analysis of IL-12/IL-23p40 transcript production was performed by real-time PCR. DCs were isolated from the spleens of *Plxnb2^−/−^* and *Plxnd1^−/−^* mice and stimulated with LPS or sham for 6 hours. Cells were harvested after 0 hours, 6 hours of sham treatment, and 6 hours of LPS treatment and IL-12 mRNA levels were determined (Figure 5 B). The data showed that IL-12/IL-23p40 mRNA levels in the wild type controls were low at the 0 and 6 hours of sham treatment time points and increased at the 6 hour with LPS treatment time point. However, IL-12/IL-23p40 expression was upregulated in *Plxnb2^−/−^* and *Plxnd1^−/−^* DCs in all time points regardless of the absence of presence of LPS stimulation. The highest level of IL-12/IL-23p40 detected was 6 hours post-LPS treatment for both Plxnb2−/− and Plxnd1−/ − DCs.

## Discussion

This study characterized the expression of Plexin-B2, Plexin-D1, and Semaphorin-3E in various immune cell types. Plexin-B2 is expressed early in development of BMDCs, decreases, and then increases at maturation and with treatment of TNFα or LPS but not TLR ligands. It is also highly expressed by pDCs. The bi-modal expression pattern in cDCs and high expression in pDCs of Plexin-B2 is opposite that of Plexin-D1. Plexin-D1 expression increases in myeloid DCs throughout maturation, and is not expressed on pDCs. Expression of Plexin-D1 by myeloid DCs is increased when these cells are activated in the presence of TLR agonists. Expression studies of a ligand for Plexin-B2 and Plexin-D1, Semaphorin-3E, revealed that Semaphorin-3E is highly expressed by Th2-type T cells and DCs. The differential expression of Plexin-B2 and Plexin-D1 on DCs in this study and previous studies in the literature demonstrating a role for Plexin-D1 in thymocyte development [12], suggests that absence of Plexin-B2 and/or Plexin-D1 would lead to abnormal T cell-DC interactions. Although a role for Plexin-D1 in the thymocyte migration has been previously reported, these studies do not address the role of Plexin-D1 in T cell activation during immune responses [12]. Studies of other plexins have demonstrated that plexins can have a profound impact on T cell-DC interactions. For example, Plexin-A1 deficient DCs result in an 85% reduction of T cell proliferation in response to antigen both *in-vitro* and *in-vivo* [14], [17].

Another recent study reported expression of Plexin-D1 and Plexin-B2 in the lungs of OVA stimulated animals [36]. Consistent with our results, in this study Plexin-B2 was expressed by lung APC-like cells further pointing to a potential role for Plexin-B2 in antigen presentation during inflammation. Plexin-D1 expression in the lung was primarily localized to the endothelial cell compartment as well as macrophages. These studies further demonstrated a role for Plexin-D1 and Plexin-B2 in inflammation through antigen presentation.

The analysis reported in this study of *Plxnb2^−/−^* and *Plxnd1^−/−^* mice did not reveal a role for Plexin-B2 or Plexin-D1 in antigen uptake by DCs or stimulation of T cells leading to proliferation. These findings suggest that while other plexins are required for T cell proliferation, Plexin-B2 and Plexin-D1 likely participate in other functions of DCs. Alternatively, there may have been other molecules that performed functions redundant to those of Plexin-B2 and Plexin-D1, which obscured the effect of individual gene deletion.

Plexins and semaphorins mediate cell migration in the immune system. Plexin-C1 is expressed on DCs and facilitates inhibition of chemokine induced migration when bound to ligand [37]. Plexin-A1 is required for transmigration of DCs across lymphatic endothelial cells yet is dispensable for chemokine induced migration in-vitro [18]. DCs migrate towards the lymph node homing chemokines CXCL12 and CCL19 upon maturation. The studies presented here show that Plexin-B2 and Plexin-D1 do not play a detectable role in migration towards lymph node homing cytokines. These studies also demonstrate that in steady state DC cell number and pattern in the spleen are similar between *Plxnb2^−/−^*, *Plxnd1^−/−^*, and wild type. DCs are very dynamic in their migration patterns throughout maturation and activation *in vivo,* and this cannot necessarily be mimicked *in vitro*. Future studies should address a role for Plexin-B2 and Plexin-D1 migration *in vivo*. Studies *in vivo* may reveal that Plexin-B2 and Plexin-D1 are involved in migration under specific conditions such as activation by pathogen or inflammatory immune environments.

To further investigate the roles of Plexin-B2 and Plexin-D1 in DC activation, cell surface markers and cytokine production by *Plxnb2^−/−^* and *Plxnd1^−/−^* DCs were assayed. The data show that Plexin-B2 and Plexin-D1 are not required for upregulation of activation markers CD40, CD80, CD86, or I-Ab in response to LPS induced activation. However, in untreated conditions and in response to LPS both *Plxnb2^−/−^* and *Plxnd1^−/−^* DCs show increased levels of IL-12/IL-23p40.

Levels of TNFα and IL-6 were not affected by lack of Plexin-B2 or Plexin-D1, indicating that only specific cytokines were affected by Plexin-B2 and Plexin-D1. Taken together, these data suggest that despite expression of Plexin-B2 and Plexin-D1 by the DC population and their upregulation post activation, these proteins are not required for expression of costimulatory molecules on the surface of DCs or for production of IL-6 and TNFα. Instead, Plexin-B2 and Plexin-D1 are required for the negative regulation of IL-12/IL-23p40 by DCs in a dramatic fashion, since the production of p40 is increased by up to 100 fold in their absence. The IL-12/IL-23p40 subunit contributes to the active forms of both IL-12 and IL-23, or can exist as a homodimer or monomer. The IL-12 pathways leads to a Th1 response, while the IL-23 pathway leads to induction of Th17 cells reviewed in [38]. Intriguingly, the IL-12/IL-23p40 subunit has been shown to exist *in-vivo* as a monomer or dimer of itself and is present in excess of IL-12 or IL-23, and is suggested to function as a negative regulator of IL-23 and/or IL-12 signaling reviewed in [39]. The monomer form of IL-12p40 can function independently. For example, the monomer form of IL-12p40 is required for dendritic cell migration in response to *Mycobacterium tuberculosis* infection [40]. In our experiments we were able to detect an increase in the mRNA levels of IL-12p35 but were unable to detect IL-23 mRNA in dendritic cells (Figure S1). Determining how IL-12 protein(s) are negatively regulated by Plexin-B2 and Plexin-D1 is a critical next step in understanding this finding and its immune consequence.

Future studies should address if both IL-12 and IL-23, which share the common p40 subunit, are affected by both Plexin-B2 and Plexin-D1. Downstream physiological consequences of overproduction of IL-12/23p40 in *Plxnb2^−/−^* and *Plxnd1^−/−^* mice, including Th skewing, response to pathogen, and potential pathways that mediate this effect should be assessed.

In summary, the studies presented here reveal that Plexin-B2 and Plexin-D1 are differentially expressed in DCs, yet surprisingly, both mediate negative regulation of IL-12/IL-23p40. These findings suggest possible crosstalk between the signaling pathways of Plexin-B2 and Plexin-D1 in DCs, similar to that previously reported in zebra fish angioblast [27]. The data suggest Plexin-B2 and Plexin-D1 function within the same pathway in DCs. The differential expression of Plexin-B2 and Plexin-D1 demonstrate that control of cell processes by plexins may be determined by their expression.

## Materials and Methods

### Ethics Statement

All studies were conducted in accordance with the National Institutes of Health Guide for the Care and Use of Laboratory Animals and were approved by the University of North Carolina Institutional Animal Care and Use Committee (protocol number 08-200).

### Mice

C57BL/6 and congenic C57BL/6 CD45.1 mice were obtained from the National Cancer Institute (Boston, MA). *Plxnd1^+/−^* mice have been previously described [11]. *Plxnb2^+/−^* mice were a gift from Dr. M. Tessier-Lavigne and have been described [35]. *Plxnb2^+/−^* and *Plxnd1^+/−^* mice were backcrossed in house at least 10 generations. OT-II mice (B6.Cg.Tg(TcraTcrb)425Cbn/J), specific for the OVA residue 323–339, were obtained from the Jackson Laboratory (Bar Harbor, ME). Mice were housed in a pathogen-free barrier facility at University of North Carolina. For fetal liver chimeras *Plxnd1* ^+/−^ or *Plxnb2^+/−^* mice were crossed for over 10 generations with C57BL/6 mice and intercrossed to obtain *^−/−^* and *^+/+^* embryos. Fetal livers were prepared from E14 embryos post PCR genotyping as previously described [11]. Fetal liver cells were injected (iv) into lethally irradiated C57BL/6 CD45.1 mice. Mice were analyzed 6–10 weeks post reconstitution. Mice were allowed to reconstitute for 6–8 weeks before use.

### ELISA

Splenic DCs were isolated from wild type, *Plxnb2^−/−^* and *Plxnd1^−/−^* animals and were stimulated for 24 hours in the presence, anti-CD40, Pam3Cys or LPS. The culture supernatants were tested for IL6, TNFα, and IL-12/IL-23p40 cytokine levels by ELISA (Ebioscience, San Diego, CA).

### Antibodies and FACS

Monoclonal Abs included: B220 (RA3-6B2), CD23 (B3B4) and APC-Alexa750-conjugated streptavidin from BD Pharmingen (San Diego, CA); CD45.2 (104), CD4 (L3T4), CD8 (Ly-2), CD3 (145-2C11), CD28 (37.51), CD11b (M1/70), CD11c (N418) and TCRβ (H57-597) from eBioscience (San Diego, CA). Secondary antibodies included anti-FITC-Alexa488 and Alexa405-conjugated streptavidin from Invitrogen (Carlsbad, California). Single cell suspensions of different tissues were counted and 10^6^ cells were suspended in FACS buffer (1xPBS plus 2% FBS) and stained with various antibody combinations. All flow cytometry was performed on a FACSCalibur and analyzed with FlowJo software (Tree Star).

### RT-PCR and Quantitative RT-PCR Analysis

RNA was isolated using a Qiagen RNA extraction kit. cDNA was synthesized using SuperScript III reverse transcriptase (Invitrogen). Primers used for RT-PCR and real-time PCR analysis were: HPRT, 5′-GCTGGTGAAAAGGACCTCT-3′, 5′-CACAGGACTAGAACA CCTGC-3′; *Plxnb2* 5′- CTAGACATCCCTGAGTCACG-3′, 5′- AGTCAGCAGTGATGCAAAGT-3′; *Plxnd1*, 5′-CCTGGGTCACCTCTGTGTTT-3′, 5′-TATCTGTCAGGCAGGGGTTC-3′; and *Semaphorin-3E*, 5′-AGGCCCTGAATACCACTGGTC-3′, 5′-GGTTCCTGTGCCAGCAAAGT-3′. Quantitative real-time PCR was performed using SYBR Green reagent in a BIORAD iCycler.

### Cell Culture

#### DC culture

Murine bone marrow DCs were isolated from wild type, *Plxnb2^−/−^*, or *Plxnd1^−/−^* mice and were cultured in the presence of GM-CSF and IL-4 as previously described [41]. *T cell culture:* T cells from OT-II mice and purified by negative selection (STEMCELL). *DC:T cell co-cultures:* DCs were harvested at day 10 and pulsed overnight with 50 µg/ml OVA (Sigma-Aldrich). 200,000 DCs were then washed and cultured in a 1∶10 ratio with T cells from OT-II transgenic T cells in 6 well plates.

### Histology

Spleens of naïve *Plxnb2^−/−^* and *Plxnd1^−/−^* mice were embedded in OCT compound, snap frozen, and stored at −80°C. 5 µm sections were prepared and fixed with 1∶1 Acetone:Methanol for 10 min at −20°C and labeled with various combinations of fluorescently labeled CD11b, CD11c, TCRβ and B220 mAb. FITC signal was amplified using anti-FITC-Alexa488 mAb. Streptavidin-AlexaFluor405 was used to amplify B220-biotin signal (blue). Images were acquired using a Zeiss LSM 710 confocal immunofluorescent microscope.

### Statistical Analysis

Statistical significance was determined with two-tailed Student’s t test or analysis of variance (ANOVA). All *p* values less than 0.05 were considered significant.

## Supporting Information

Figure S1 ***Plxnb2^−/−^*** **and** ***Plxnd1^−/−^*** **DC time-course of IL-12/p35 and IL-23 cDNA.** DCs were isolated from the spleens of wild type, *Plxnb2^−/−^* and *Plxnd1^−/−^* mice and mRNA was isolated to determine transription levels of IL-12/p35 and IL-23 by real-time PCR. Data are representative of 3 independent experiments. n = 3–4 mice per group. *p<0.01.
(TIF)
