# Supplementary figures and images for: Plexin-B2 and Plexin-D1 in Dendritic Cells: Expression and IL-12/IL-23p40 Production
Source: PLoS One. 2012 Aug 15;7(8):e43333. doi: 10.1371/journal.pone.0043333 (PMC3419716; doi:10.1371/journal.pone.0043333)

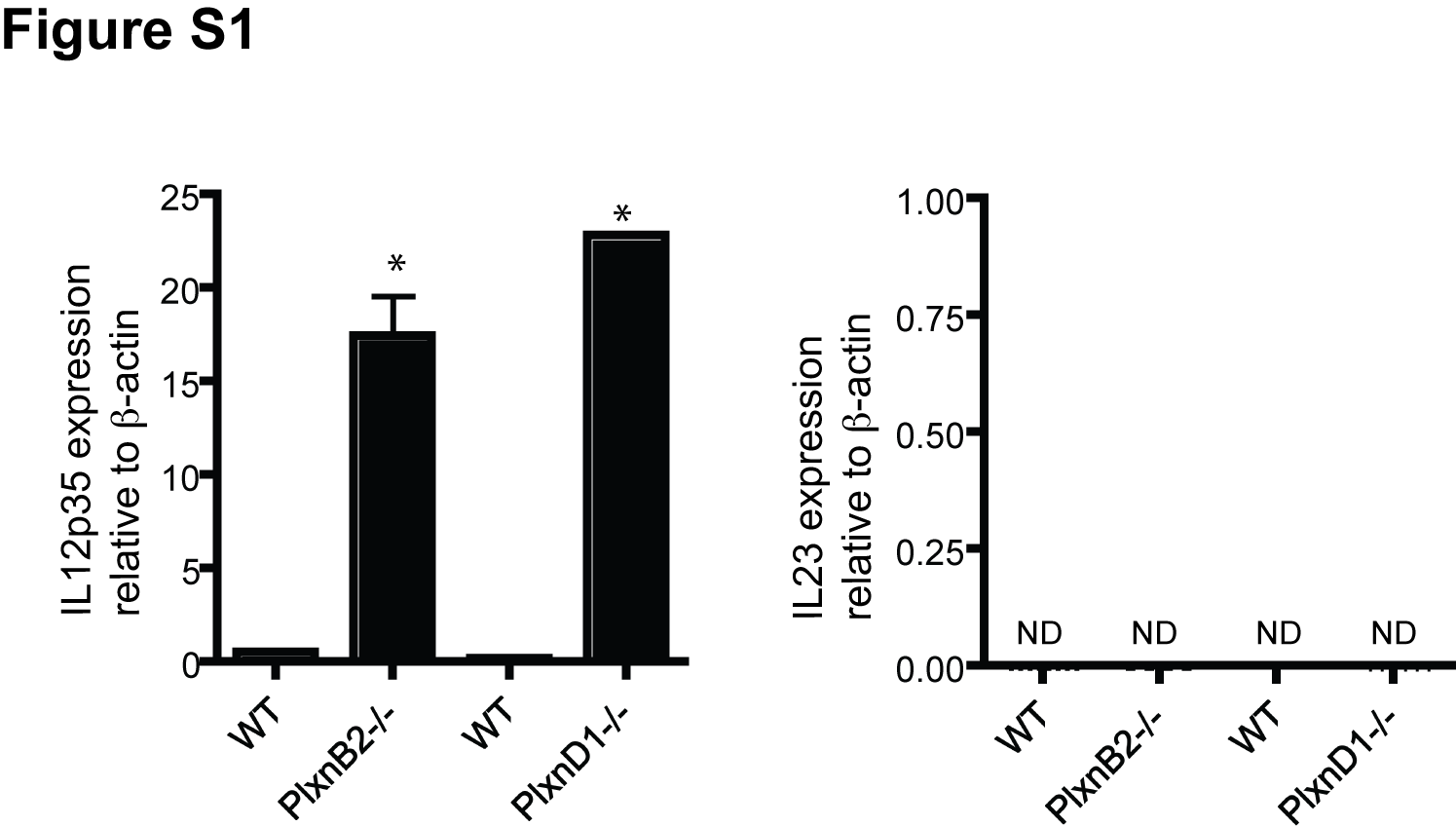

Supplement: Figure S1 — Plxnb2−/− and Plxnd1−/− DC time-course of IL-12/p35 and IL-23 cDNA. DCs were isolated from the spleens of wild type, Plxnb2−/− and Plxnd1−/− mice and mRNA was isolated to determine transription levels of IL-12/p35 and IL-23 by real-time PCR. Data are representative of 3 independent experiments. n = 3–4 mice per group. *p<0.01. (TIF) [file pone.0043333.s001.tif]
